# Exploring toothpaste ingredients as potential chemical kindling agents in neuropsychiatric disorders: An adult zebrafish (*Danio rerio*) study

**DOI:** 10.1016/j.ibneur.2026.07.008

**Published:** 2026-07-10

**Authors:** Thomas Mathew, Shagun Bhardwaj, Aditi Goyal, Astha Bhatt, Anita Mahadevan, Mohammed Shakeebuddin Kashif, Paul Bundi Karau, Devina Mitra, Binoy V V, Joby Pullikan, Joel Jose, Kowshik Kukkemane, Pritam Majumdar, Prashanth Thankachan, Raghunandan Nadig

**Affiliations:** a St. John’s Medical College Hospital, Bengaluru, India; b National Institute of Mental Health and Neurosciences, Bengaluru, India; c National Institute of Advanced Sciences, Bengaluru, India; d St John’s Research Institute, Bengaluru, India; e Department of Life Sciences, Christ (Deemed to be University), Bengaluru, India

**Keywords:** *Danio rerio*, Neurotoxicity, Anxiety, Aggression, Glial fibrillary acidic protein (GFAP)

## Abstract

**Introduction:**

Certain ingredients in toothpaste have the potential to cause headache disorders and seizures. Essential oils, particularly camphor and eucalyptus, may contribute to neuronal excitability. We hypothesize that toothpaste containing brain-stimulating substances may induce behavioral changes in zebrafish. The aim of this study was to investigate the neurobehavioral and histopathological effects of exposure to three commercially available toothpaste formulations in adult zebrafish (*Danio rerio*).

**Methods:**

We used four tanks, each containing 10 adult zebrafish, with one control and the three intervention tanks. 100 mg of three different toothpastes was added to the tanks for two weeks, after which the fish were observed for the next six weeks. The Mirror Biting Test and the Novel Tank Test were conducted at 4 weeks after intervention to assess aggression and anxiety, respectively. Python software was used to track the fish movements. Six weeks post intervention, histology and glial fibrillary acidic protein (GFAP) immunohistochemistry between the test groups were compared.

**Results:**

During the two-week screening phase prior to the addition of toothpaste, all the fish were healthy and exhibited normal activity. During the intervention, hyperactivity was observed in all intervention tanks compared to the control, especially in Tank 2, which contained a camphor-based toothpaste. The fish in the control tank moved at an average speed of 9.10 pixels/frame, while those in Tanks 1, 2, and 3 moved at 13.25 pixels/frame, 9.43 pixels/frame, and 9.94 pixels/frame, respectively. The Mirror Biting Test and the Novel Tank Test indicated increased aggression and anxiety in all intervention tanks, with the most notable effects observed in Tank 2. Histopathological examination revealed no neuronal alterations; however, an increase in GFAP expression was detected across all intervention groups.

**Conclusions:**

The findings of this study suggest that toothpaste ingredients can cause hyperactivity, aggression, anxiety and astrocytosis in adult zebra fish.

## 1 Introduction

Certain ingredients in toothpaste like fluoride, sodium benzoate, pentasodium phosphate, camphor, eucalyptus etc may have stimulant effects on neurons and have the potential to cause seizures, headache, anxiety and behavioural changes (Mathew et al., 2017, Mathew et al., 2021, Mathew and John, 2020, Mathew et al., 2020, Mathew et al., 2022). Pulsatile exposure to chemicals every 24–48 h is known to cause chemical kindling (Goddard, 1983). Kindling may be the mechanism underlying the pathogenesis of hyperexcitable neuropsychiatric disorders like epilepsy and anxiety. We hypothesize that toothpaste being used every 24 h may be an agent for chemical kindling. To test this hypothesis, we used zebrafish (*Danio rerio*), a well recognized model for translational neuroscience and behavioural research. We exposed adult zebrafish to commonly used toothpastes and analysed their behaviour and histopathological changes in the brain post exposure. Here we report the findings of this study.

Certain ingredients in toothpaste like fluoride, sodium benzoate, pentasodium phosphate, camphor, eucalyptus etc may have stimulant effects on neurons and have the potential to cause seizures, headache, anxiety and behavioural changes (Mathew et al., 2017, Mathew et al., 2021, Mathew and John, 2020, Mathew et al., 2020, Mathew et al., 2022). Repeated intermittent exposure to neuroactive chemicals can induce a phenomenon known as chemical kindling, whereby subthreshold stimuli administered at regular intervals progressively enhance neuronal excitability (Goddard, 1983). The ideal time of the delivery of the stimulus for this kindling to take effect is every 24–48 h and many of the day-to-day personal hygiene products such as toothpaste are used at intervals of 24 h, making them an ideal agent of chemical kindling. Chemical kindling has been proposed as a mechanistic basis for the development of hyperexcitable neuropsychiatric disorders, including epilepsy and anxiety disorders.

Toothpaste ingredients can get absorbed through the highly vascular buccal mucosa and can reach systemic circulation and the brain bypassing the first-pass metabolism in the liver. Although the quantity absorbed during a single exposure is generally low, the cumulative neurobiological consequences of repeated exposure may be significant and have not been adequately investigated. We therefore hypothesized that chronic daily exposure to commercially available toothpastes may act as a chemical kindling stimulus, leading to progressive alterations in neuronal excitability that manifest as behavioural abnormalities and histopathological changes in the brain.

To test this hypothesis, we employed zebrafish (*Danio rerio*), a well-established model in translational neuroscience and behavioural research. Adult zebrafish were exposed to commonly used toothpastes in a regular, repetitive manner, and behavioural responses and brain histopathological changes were assessed following exposure. Here, we report the findings of this study.

### 1.1 Objective

To evaluate the effect of three commonly used toothpaste on adult zebrafish with respect to anxiety, aggression and pathological changes in the brain.

## 2 Materials and methods

### 2.1 Zebrafish husbandry

Adult wild type zebrafish (*Danio rerio*), 6–8 months old, of both sexes were procured from the Fisheries Research and Information Centre of the Karnataka Veterinary, Animal and Fisheries Sciences University and maintained under standard laboratory conditions of 28°C with a 14:10 h light-dark cycle. The fish were fed once daily with commercial pellets. All experiments were performed at the St. John’s Medical College Animal House following the Institutional Animal Ethics Committee (IAEC) and CPSCEA guidelines (IAEC Ref. No. 01/2024). A total of 10 fish were housed in a 20 L tank and a uniform stock density was maintained.

### 2.2 Exposure to toothpaste

A total of 40 adult zebrafish were randomly assigned to four groups (n = 10 fish per group). Group I served as the control and was maintained in tank water without toothpaste exposure. Groups II, III, and IV were exposed to toothpaste−1, toothpaste−2, and toothpaste−3, respectively. Each group was housed in a separate 20 L tank with a stocking density of 10 fish per tank. Following four weeks of exposure, all fish were subjected to behavioural assessments, including the Novel Tank Diving Test and Mirror-biting Test. Subsequently, fish were maintained under their respective exposure conditions until six weeks, after which brain tissues were collected for histological and immunohistochemical analyses.

### 2.3 Behavioural paradigms

#### 2.3.1 Novel tank diving test

This test is used to assess anxiety-like behaviour in zebrafish. The experimental setup comprised of a 20 L tank of dimensions 20 cm length, 30 cm and 36 cm (height). The tank was divided into a top and bottom zone virtually. The fish was individually placed in the tank and was allowed to swim freely for 5 min. The movements were recorded using a 12 mega pixel, 4 K HD camera. After performing the paradigm, the fish were returned to their respective exposure tanks. Videos were analysed for velocity and trajectory of movements by using object detection architecture software (YOLOv11) and DeepLabCut, an animal pose estimation software for behavioural studies.

#### 2.3.2 Mirror-biting test

This test is used to assess aggression-like behaviour in zebrafish. The experimental setup comprised of a 20 L tank of dimensions 20 cm length, 30 cm and 36 cm (height). The fish was individually placed in the tank with a mirror placed horizontally against the wall. The movements were recorded using a 12 mega pixel, 4 K HD camera. After performing the paradigm, the fish were returned to their respective exposure tanks. Video recordings were analyzed to quantify locomotor parameters, including velocity and movement trajectories, using object detection architecture software (YOLOv11) and DeepLabCut, a marker less animal pose estimation software widely used for behavioral analysis. Aggressive behavior was further quantified by manually counting the number of mirror-directed biting events.

### 2.4 Immunohistochemistry

After 6 weeks, the fish were euthanised by immersing them in ice-cold water for approximately 10 s. The head region was then dissected and fixed in formalin overnight. Post fixation, the tissue was processed and embedded in paraffin blocks. Sections taken were stained with hematoxylin and eosin (H&E) and luxol fast blue for histological analysis. Additionally, immunohistochemistry for glial fibrillary acidic protein (GFAP) (Zeta Corp, GA5, RTU) was done and analysed. The histology and GFAP immunohistochemistry between the control and test groups were compared.

## 3 Results

During the screening phase, all the fish were healthy and normally active. All zebrafish underwent a health screening assessment during acclimatization. Fish were considered healthy based on normal external morphology (intact fins, absence of lesions, normal body shape, and absence of discoloration), active swimming behavior, appropriate response to visual and tactile stimuli, and normal feeding behavior. Fish displaying lethargy, abnormal buoyancy, erratic swimming, visible deformities, or reduced feeding response were excluded from the study. The three commonly used toothpastes and their ingredients are shown in Table 1. By the third day of the intervention, hyperactivity was observed in all three tanks with maximum hyperactivity noted in tank 2.

**Table 1:** Ingredients in the three commonly used toothpastes.

	**Toothpaste 1**	**Toothpaste 2**	**Toothpaste 3**
Ingredients	Maricha(Piper nigrum)	Hydrated Silica	Precipitated Silica
	Pippali (Piper longum)	Titanium Dioxide	Titanium Dioxide
	Shunthi (Zingiber officinale)	Sodium Monofluorophosphate	Sodium Fluoride
	Tomar (Zanthoxylum armatum)	Calcium carbonate	Sodium Saccharin
	Lavanga (Syzygium aromaticum)	Sodium Lauryl Sulfate	Sorbitol
	Karpura (Cinnamomum camphora)	Flavour	Sodium Propyl Hydroxybenzoate
	Pudina (Mentha species)	Potassium Nitrate	Sodium Methyl Hydroxybenzoate
	Gairic powder	Sodium Bicarbonate	Sodium Methyl Cocoyl Taurate
	Sodium Saccharin	Limonene	Xanthan Gum
	Sodium Benzoate	Sodium Saccharin	Glycerol
		Sorbitol	Strontium Acetate
		Sodium Silicate	
		Benzyl Alcohol	
		Carrageenan	
		Arginine	

During the weekly evaluation, the hyperactivity that was observed in all the three tanks persisted and was most obvious in tank 2. Velocity measurement showed increased velocity in Tank 2 (13.25 pixels/frame) in comparison to all other tanks (control - 9.10 pixels/frame; tank 3–9.43 pixels/frame; tank 4–9.94 pixels/frame) (Table 2).

**Table 2:** Velocity of fish in the control and intervention tanks.

	**Tank 1** ***(control)***	**Tank 2*****(toothpaste 1)***	**Tank 3*****(toothpaste 2)***	**Tank 4*****(toothpaste 3)***
**Velocity**	9.10	13.25	9.43	9.94

The novel tank test done showed fish in the control tank spent more time in the upper part of the tank while those in the intervention tanks spent most of their time at the bottom suggesting that fish in the intervention tank were anxious. The time spent by the fish in each tank is shown in Fig. 1 and the trajectory of exploration of fish in the tanks during the novel tank test is shown in Fig. 2.

**Fig. 1:**
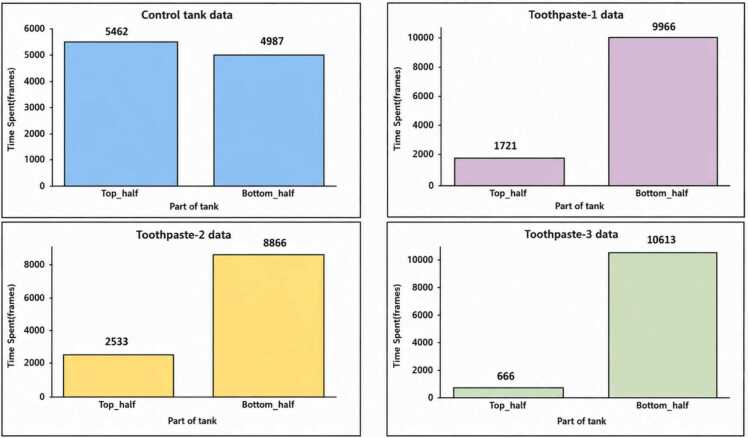
Representative Novel Tank Diving Test analysis from one zebrafish from each experimental tank. The graphs depict the total time (frames) spent in the upper and lower halves of the tank by a single representative zebrafish from the control, Toothpaste−1, Toothpaste−2, and Toothpaste−3 groups. These data are presented as representative behavioural tracking and are not summary statistics of all animals in each group.

**Fig. 2:**
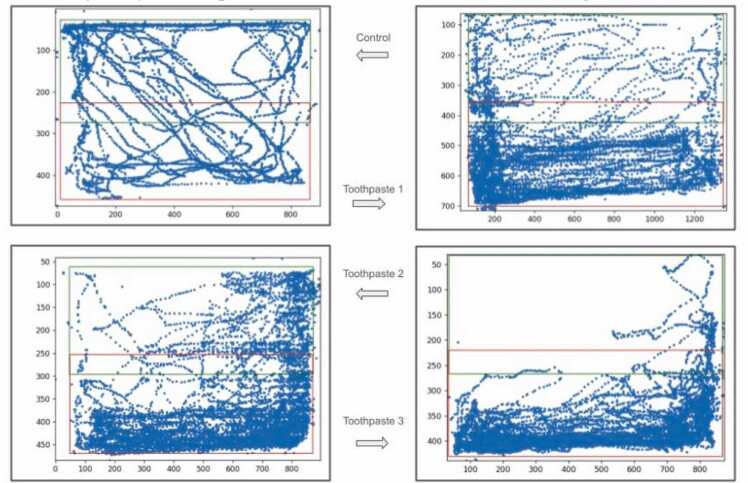
Trajectory of exploration of fish in the tanks during the novel tank test.

In the mirror biting test, fish in Tank 2 were aggressive and repeatedly attacked the mirror when compared to the other tanks and advances to the mirror was least observed in the control tank. However, we did not have any software to assess these movements.

Post intervention, during the observation phase, one zebrafish died in the control tank on day 11 and 6 zebrafish died in the control tank on day 21. We checked the ammonia levels in the tanks to identify the cause of mortality in the control tanks and we found that ammonia levels were markedly elevated in the control tank compared to the other three tanks. The ammonia levels of individual tanks are given in Table 3. We also took water for a microbiological investigation and found that there was a heavy growth of *Pseudomonas aeruginosa* in the control tank.

**Table 3:** Levels of ammonia in control and intervention tanks.

	**Tank 1*****(control)***	**Tank 2**	**Tank 3**	**Tank 4**
**pH**	5.93	8.06	8.18	7.81
**Ammonia**	124.79	4.77	< 0.00	33.37
*Pseudomonas aeruginosa*	++++	+	+	+

The histopathological examination showed no neuronal alterations on H&E between the control and test groups. There were no hypoxic changes in the neurons. Reactive hypertrophic astrocytes were not seen. There was no significant inflammation. Luxol fast blue highlighted preserved myelin. On immunohistochemistry, there was a prominent increase in the GFAP expression in all the intervention groups compared to control (Fig. 3).

**Fig. 3:**
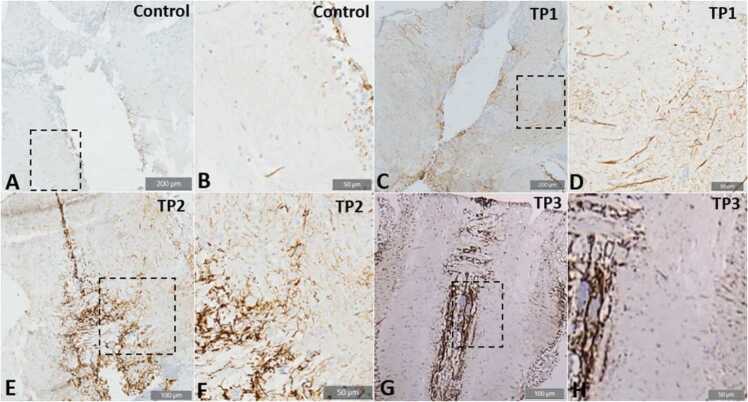
Immunohistochemistry for glial fibrillary acidic protein (GFAP) in control and toothpaste-treated zebrafish. **A, C, E, G:** low power photomicrographs; **B, D, F, H:** higher magnification views of the boxed regions in the corresponding adjacent panels. **A-B**: Control zebrafish showing sparse GFAP-immunoreactive glial fibers. **C-H:** Treated zebrafish exposed to **toothpaste 1 (C-D), toothpaste 2 (E-F), toothpaste 3 (G-H)** demonstrating increase in GFAP positive glial fibers compared with controls, indicating reactive gliosis (astrogliosis) in these groups. [Magnification= Scale bars: A, C – 200 μm; E, G – 100 μm; B, D, F, H – 50 μm].

## 4 Discussion

Our preliminary experiment on commonly used toothpastes on adult zebrafish suggest that toothpaste ingredients may have an impact on the behaviour, glial density and may affect the gut microbiome of the fish. The zebrafish exposed to toothpaste showed hyperactivity and anxious behaviour when compared to the control. The effects were variable in the different tanks. The fish in tank 2 exposed to toothpaste 1 which had ingredients like camphor, eugenol (component of lavanga), and menthol, showed higher levels of anxiety based on the novel tank test. Histopathological evaluation showed increased astrocytosis in the brain of the zebrafish exposed to all the three toothpastes.

The behavioural phenotype observed in the present study, characterized by increased locomotor activity, anxiety-like behaviour in the novel tank test, and increased aggression in the mirror-biting test, is broadly consistent with behavioural alterations reported following exposure to several individual toothpaste constituents. Previous zebrafish studies have demonstrated anxiety-like behaviour, altered locomotion, oxidative stress, and neurodevelopmental toxicity following exposure to fluoride, sodium benzoate, camphor, silica nanoparticles, titanium dioxide nanoparticles, and other environmental neurotoxins. Collectively, these findings support the possibility that the behavioural abnormalities observed in our study may arise from additive or synergistic effects of multiple neuroactive ingredients present within commercial toothpaste formulations rather than from a single constituent alone.

A number of ingredients in toothpaste are implicated in neuronal dysfunction and hyperexcitability. In a study by Karaman et al. (2023) exposure to varying amounts of fluoride was shown to disrupt circadian rhythms, induce anxiety-like behaviour, decrease melatonin levels, increase oxidative stress, and structural brain alterations. Fluoride also promotes excitotoxicity by inhibiting acetylcholinesterase and glutamate uptake (Dondossola et al., 2022). Fluoride, in a dose dependent manner, is known to alter gene expression in zebrafish (Wang et al., 2025). Emerging evidence also suggests change in gut-brain axis in fluoride-exposed zebrafish (Jin et al., 2024).

Another ingredient sodium benzoate is known to increase thigmotaxis, a "wall-hugging" or "edge-hugging" behavior an indicator of anxiety and stress in zebrafish (Chen et al., 2009). Sodium benzoate was tested using lower concentrations than the safety limits and was revealed to induce developmental defects, anxiety like behaviour and mortality. They observed an upregulation of glutathione reductase as a response to exposure to sodium benzoate, indicating increased oxidative stress in the zebrafish larvae (Gaur et al., 2018). A similar study on the effect of silica nanoparticles (SiO2 NPs) showed an increase in depression and anxiety and inhibited learning and memory in zebrafish (Li et al., 2020). SiO2 NPs result in dose-dependent neurotoxic effects, associated with changes in locomotor activities and anxiety behaviours. One study showed a biphasic alteration, with reduced locomotion after 24 h of exposure and hyper-locomotion after a 48-hour exposure (Shen et al., 2025). Titanium dioxide (TiO2) nanoparticles are known to cross the blood brain barrier and accumulate in the neural tissues (Zhu et al., 2023). TiO_2_ is also considered as one of the most prevalent nanoplastic in the aquatic environment (Buzenchi Proca et al., 2024). Exposure to titanium nanoparticles has shown to increase anxiety-like responses, alter spatial recognition memory and motor activity and cause changes in social behaviours in both larvae and adult zebrafish (Chen et al., 2021). In a study investigating the mechanism of TiO_2_ induced neurotoxicity, researchers found that norepinephrine, dopamine, and 5-hydroxytryptamine were significantly decreased and nitric oxide (NO) levels were markedly elevated, over proliferation of glial cells, and neuron apoptosis was seen (Sheng et al., 2016a). Other ingredients like nitrates and nitrites, are reported to increase anxiety-like behaviour and decrease associative learning and executive function (Beaver et al., 2020). Nitrate also causes a decrease in brain GABA levels, an inhibitory neurotransmitter resulting in neuronal hyperexcitability. It can also produce oxidative stress, neuroinflammation and apoptosis (García-Jaramillo et al., 2020). Sodium lauryl sulphate, another commonly prevalent ingredient in toothpaste, is known to induce anxiety in zebrafish at low concentrations (Impellitteri et al., 2022). Saccharin, an artificial sweetener, is also known to cause neurobehavioural changes including anxiety like behaviour and metabolic syndrome in adult zebrafish (Han et al., 2021, Kumaran, 2025).

Many of the herbal ingredients in the toothpaste 1 might have contributed to neuronal hyperexcitability. Eugenol in lavanga, can induce central nervous system hyperstimulation, neuronal excitability and seizure-like activity as confirmed by EEG (Barbas et al., 2021). Camphor in toothpaste 1 is also known to cause neurotoxicity, behavioural alterations, and molecular, and genetic changes in zebrafish. 4-Methylbenzylidene camphor is known to alter the gene expression involving shha, ngn1, mbp, elavl3, α1-tubulin, syn2a, and gap43 genes and interfere with sonic hedgehog signaling (Prakash et al., 2024). Exposed embryos exhibit significant thigmotaxis.‌ Sub-lethal doses of camphor triggered cardiotoxicity, oxidative stress, and apoptosis, marked increase in reactive oxygen species (ROS) and malondialdehyde (MDA), and decreased superoxide dismutase (SOD) activity (Du et al., 2021). These results suggest camphor’s developmental toxicity may be linked to oxidative stress and cell death mechanisms. Menthol, another ingredient in the toothpaste, alters key brain enzymes like acetylcholinesterase and tyrosine hydroxylase, leading to anxiety-like behaviour (Kumaran, 2025). It also interferes with calcium signaling, activates the Nrf2 pathway and increases oxidative stress resulting in abnormal nerve function (Carneiro et al., 2022). Oxidative stress, if unregulated, can alter the immune system, prolong inflammation and also contribute to ageing (“inflammaging”) (Bajaj et al., 2025). We have also observed astrocytosis in the brain of zebrafish exposed to toothpaste in the current study similar to previous studies which had found astrocytosis in the zebrafish following exposure to silica and titanium dioxide nanoparticles (Sheng et al., 2016b, Li et al., 2021).

Although we have not studied gut bacteria, a change in the levels of ammonia in the tanks may indicate possible gut dysbiosis. In tanks with zebrafish, ammonia levels accumulate due to excretion of ammonia as a waste product of protein metabolism. Ammonia can be directly released across the gills and is excreted through urine and faeces. In a closed experimental set up without water exchange, as in this study, ammonia gradually builds up. Adult zebrafish of 0.4–0.6 g each typically excrete 0.2–0.5 mg of ammonia nitrogen per gram of fish per day. 10 fish (approximately 5 g) produces (5 ×0.3 mg/g/day) 1.5 mg of ammonia per day. Over 21 days in a 20 L tank, there would be approximately 1.6 mg/L of NH3-N in the tank (∼ 117.5 μmol/L NH3-N in the tank). Our control tank had 124.79 μmol/L. But other tanks had much lower ammonia levels ranging from 0 to 33.37 μmol/L suggesting a role of toothpaste ingredients reducing ammonia production in the fish, most probably by gut dysbiosis. There is a possibility that increased ammonia levels in the control tank may have dulled the behavior of the zebrafish in that tank. However, fish in Tank 2 (ammonia level - 4.77 μmol/L) exhibited greater behavioral abnormalities compared to fish in Tank 3, despite Tank 3 having lower ammonia levels (0.0 μmol/L). Ingredients in toothpaste that have the potential to cause gut dysbiosis are fluoride, titanium dioxide, nitrate, and sodium benzoate (García-Jaramillo et al., 2020, Chen et al., 2025, Chen et al., 2018, Tsay et al., 2007).

We embarked on this study based on our observation in patients that cessation of toothpaste exposure resulted in improvement in anxiety and headaches. In this study, we observed that adult zebrafish exposed to three commonly used toothpastes showed changes in behaviour, especially hyperactivity, anxiety, and aggression. We also observed increased GFAP expression in toothpaste exposed zebrafish brains and not in the controls. This increased GFAP expression suggests astroglial activation likely in response to neural stress. Astrocytosis is increasingly recognized as an early indicator of neuroinflammation and neural injury in toxicological models, often preceding overt neuronal loss. The absence of detectable neuronal degeneration on routine histopathology, together with increased GFAP expression, may therefore indicate an early or subclinical neurotoxic response rather than established structural brain injury (Zheng et al., 2024).

The strengths of this study are that we have used commonly used toothpaste and have administered it in a pattern similar to human exposure and showed the impact on behaviour, glial density, and indirect changes in gut microbiome of adult zebrafish. Toothpaste with various ingredients which can cross the blood brain barrier may excite neurons on a regular basis in a sub-clinical way and has the potential to chemically kindle the human brain. This chemical kindling can cause changes in neuronal circuitry and hyperexcitability and may result in various neurological and psychiatric disorders.

The present study has several limitations. First, we evaluated the effects of whole toothpaste formulations rather than individual constituents; therefore, it is not possible to identify which specific ingredient or combination of ingredients was responsible for the observed behavioural and histopathological changes. Second, although toothpaste was added daily at standardized amounts, we did not quantify the concentrations of individual compounds in the tank water over time. Consequently, biological uptake, adsorption to tank surfaces, degradation, or interactions among ingredients may have influenced the actual exposure levels experienced by the fish. Third, we were unable to assess biochemical markers of stress, neuroinflammation, oxidative stress, or neuronal injury, such as cortisol levels and oxidative stress biomarkers, which could have provided mechanistic insights into the observed behavioural changes and increased GFAP expression. Fourth, differences in water quality parameters, including ammonia accumulation, bacterial overgrowth, and mortality in the control tank, may have influenced behavioural outcomes and therefore represent potential confounding factors. However, the behavioural abnormalities across the intervention tanks (especially tank 2 showing hyperactivity compared to tank 3 and 4) and histopathological changes in the brain of the exposed fish shows an effect of toothpaste on the brain and behaviour of zebrafish. The addition of whole toothpaste in the closed aquatic system might have created an undefined environment making scientific conclusions impossible. However, human beings are subjected to a similar concoction of toothpaste ingredients daily. Hence, we designed a model to mimic a similar exposure as human beings. Literature also has plenty of evidence showing that the ingredients of toothpaste have neurotoxic effects on both adult and larval zebrafish.

The findings of this exploratory study warrant further investigation into the neurobehavioral effects of toothpaste ingredients. Future studies should evaluate individual toothpaste constituents and their combinations to identify compounds that may contribute to neuronal hyperexcitability and behavioural alterations. Dose-response studies, measurement of exposure concentrations, and assessment of biomarkers related to oxidative stress, neuroinflammation, neurotransmitter alterations, and stress responses would help clarify underlying mechanisms. Given the observed increase in GFAP expression, future work should also include detailed molecular and immunohistochemical analyses of neural injury and glial activation. In addition, microbiome profiling may help determine whether alterations in gut microbial communities contribute to behavioural changes through gut-brain interactions. Replication of these findings in other experimental models and under exposure conditions that more closely mimic human use patterns will be important to establish the translational relevance of the observations. While the present study does not establish adverse neurological effects in humans, it highlights the need for further research into the long-term neurobiological consequences of repeated exposure to complex chemical formulations.

## 5 Conclusions

In this study, we observed that toothpaste ingredients may induce hyperactivity, anxiety-like behaviour, and aggression in adult zebrafish. We also found that exposure to toothpaste formulations was associated with astrocytosis in the zebrafish brain and possible alterations in the gut microbiome. The implications of these findings for humans require further investigation. If similar effects are demonstrated in humans, they may provide insights into the pathogenic mechanisms underlying various neuropsychiatric disorders, like migraine, epilepsy, anxiety disorders, and autism spectrum disorder.

## CRediT authorship contribution statement

**Shagun Bhardwaj:** Writing – review & editing, Writing – original draft, Visualization, Resources, Methodology, Investigation, Formal analysis, Data curation. **Aditi Goyal:** Writing – review & editing, Writing – original draft, Visualization, Validation, Software, Resources, Methodology, Investigation, Formal analysis. **Thomas Mathew:** Writing – review & editing, Writing – original draft, Visualization, Validation, Supervision, Resources, Project administration, Methodology, Investigation, Formal analysis, Data curation, Conceptualization. **Joby Pullikan:** Writing – review & editing, Visualization, Supervision, Investigation, Formal analysis. **Joel Jose:** Writing – review & editing, Visualization, Supervision, Project administration, Methodology. **Devina Mitra:** Writing – review & editing, Writing – original draft, Visualization, Formal analysis. **V V Binoy:** Writing – review & editing, Visualization, Supervision, Software, Formal analysis. **Mohammed Shakeebuddin Kashif:** Writing – review & editing, Writing – original draft, Visualization, Software, Formal analysis. **Karau Paul:** Writing – original draft, Visualization, Validation, Formal analysis. **Anita Mahadevan:** Writing – review & editing, Writing – original draft, Visualization, Validation, Software, Resources, Methodology, Investigation, Formal analysis. **Astha Bhatt:** Writing – review & editing, Writing – original draft, Visualization, Software, Methodology, Formal analysis, Data curation. **Prashanth Thankachan:** Writing – review & editing, Validation, Supervision, Project administration, Methodology, Formal analysis. **Raghunandan Nadig:** Writing – review & editing, Supervision, Resources, Project administration, Methodology. **Kowshik Kukkemane:** Writing – review & editing, Visualization, Supervision, Project administration, Methodology. **Pritam Majumdar:** Writing – review & editing, Writing – original draft, Visualization, Investigation, Formal analysis.

## Compliance with ethical standards

All experiments were performed at the St. John’s Medical College Animal House following the Institutional Animal Ethics Committee and CPSCEA guidelines. A total of 10 fish were housed in a 20 L tank and a uniform stock density was maintained.

## Funding sources

This research did not receive any specific grant from funding agencies in the public, commercial, or not-for-profit sectors.

## Declaration of Generative AI and AI-assisted technologies in the writing process

During the preparation of this work the author(s) used Google Notebooklm to generate the graphical abstract based on the study and its results. The final outcome of the tool/service was reviewed and edited by the author(s) as required and the author(s) therefore take(s) full responsibility for the content of the published article.

## Declaration of Competing Interest

The authors declare that they have no conflicts of interests.

## Data Availability

Researchers who wish to access our data may send an email to the corresponding author.
